# F118. ARCHITECTURE OF PSYCHOSIS SYMPTOMS AND NEURAL PREDICTORS OF CONVERSION AMONG CLINICAL HIGH RISK INDIVIDUALS WITH AUTISM SPECTRUM DISORDER

**DOI:** 10.1093/schbul/sby017.649

**Published:** 2018-04-01

**Authors:** Jennifer Foss-Feig, Eva Velthorst, Sylvia Guillory, Holly Hamilton, Brian Roach, Peter Bachman, Aysenil Belger, Ricardo Carrion, Erica Duncan, Jason Johannesen, Gregory Light, Margaret Niznikiewicz, Jean Addington, Kristin Cadenhead, Tyrone Cannon, Barbara Cornblatt, Thomas McGlashan, Diana Perkins, Larry Seidman, Ming Tsuang, Elaine Walker, Scott Woods, Carrie Bearden, Daniel Mathalon

**Affiliations:** 1 Icahn School of Medicine at Mount Sinai; 2 University of California, San Francisco, San Francisco VA Health Care System; 3 Northern California Institute for Research and Education; 4 University of Pittsburgh; 5 University of North Carolina; 6 Zucker Hillside Hospital; 7 Emory University, Atlanta VA Medical Center; 8 Yale University, VA Connecticut Healthcare System; 9 University of California, San Diego; 10 Harvard Medical School/BHCS; 11 University of Calgary; 12 Yale University; 13 Harvard Medical School; 14 Emory University; 15 University of California, Los Angeles

## Abstract

**Background:**

Individuals with autism spectrum disorders (ASD) have symptoms, including social and sensory deficits, and neurobiological alterations that overlap with schizophrenia. Though there is evidence of high rates of psychosis symptoms in ASD, little is known about psychosis prodrome in ASD, or about predictors of psychosis conversion in this population. In this study, we leverage data from clinical high risk (CHR) patients from the NAPLS2 consortium to examine: a) baseline differences in psychosis symptoms and social functioning, b) relative risk of conversion, and c) whether neural response to sensory stimuli yields differential predictors of conversion in CHR individuals with and without ASD (CHR/ASD+; CHR/ASD-).

**Methods:**

Clinical, electrophysiological, and 24-month follow-up data were available for 305 individuals (14 CHR/ASD+; 291 CHR/ASD-). We examined baseline differences on the SOPS, GFS, and TASIT. Conversion risk was computed with the Cannon conversion calculator, and conversion was defined as SOPS>6 at 2-year outcome. P300 event-related potentials (ERP) were extracted from ongoing EEG collected at baseline in response to Target and Novel auditory and visual stimuli, each presented on 10% of trials within streams of 80% standard stimuli in the same modality.

**Results:**

In line with our expectations, CHR/ASD+ had worse functioning than CHR/ASD- on the GF-Social scale (t=-4.2, p<.01) and TASIT total score (t=-2.9, p=<.01), but groups did not differ in their psychotic symptoms on the SOPS (Positive: p=.72; Negative: p=.13; Disorganization: p=.13; General: p=.86). Groups did not differ in the rate at which they converted to psychosis (CHR/ASD+: 15.4%; CHR/ASD-: 11.1%; p=.50), and the Cannon risk score was equally predictive of 2-year conversion across groups (p=.39). EEG data revealed dissociable profiles regarding neural response to sensory stimuli in those who did versus did not convert to psychosis, depending on ASD status. P300 response over central electrodes to Novel visual stimuli was weaker in CHR- converters (n=71) than CHR- non-converters (n=220), but stronger in CHR/ASD+ converters (n=4) than CHR/ASD+ non-converters (n=10) (Novel Stimuli: Modality by ASD interaction, F=5.66, p=.02; Modality by ASD by Converter Interaction, F=3.57, p=.06). For both auditory and visual Target stimuli, P300 response over parietal electrodes did not differ between CHR/ASD- converters and non-converters; however, whereas CHR/ASD+ individuals who did not convert had amplitudes similar to all CHR/ASD- individuals, CHR/ASD+ converters had substantially greater auditory and visual P300 amplitudes (Target Stimuli: ASD by Converter interaction, F=12.12, p=.001).

**Discussion:**

Individuals with ASD and CHR have greater social deficits than the general CHR population, but show similar psychotic symptoms and have similar risk for conversion to psychosis. Neural response to sensory stimuli is important for understanding risk for conversion, and differs among CHR individuals dependent on whether they have ASD. In particular, whereas all CHR individuals who do not convert share a common pattern of attenuated ERP amplitudes reflecting attention allocation to target and novel auditory and visual stimuli, CHR/ASD+ who convert have a unique pattern of globally heightened P300 responses to infrequent novel and target stimuli. These findings have two important implications: 1) individuals with ASD do convert to psychosis and have similar CHR symptom and risk profiles to non-ASD CHR patients clinically; 2) in CHR individuals with ASD in particular, examining neural markers of attention allocation to sensory stimuli may reveal important predictive clues about risk for conversion.

